# Correction to “Analysis of the clinical and genetic characteristics of a Chinese family with osteogenesis imperfecta type I”

**DOI:** 10.1002/mgg3.2497

**Published:** 2024-08-12

Niu, Z., Lai, Y., Zhou, W., Liu, L., Tan, S., He, G., Li, J., Tang, F., Su, Y., Xu, Y., Liu, L., Xie, L., Fang, Q., & Tang, A. (2022). Analysis of the clinical and genetic characteristics of a Chinese family with osteogenesis imperfecta type I. *Molecular Genetics & Genomic Medicine*, 10, e2019. https://doi.org/10.1002/mgg3.2019

In the originally published article, there was a discrepancy between the order of the funders listed on the first page of the article and in the Acknowledgments section. The correct order is: Specialized Research Fund for Young and Innovative Scholarship, Grant/Award Number: AD20297002 and AD19245007; National Natural Science Foundation of China, Grant/Award Number: 82101232, 8176040078, and 82160217; China Postdoctoral Science Foundation, Grant/Award Number: 2019M663875XB and 2019M663413. This has been updated in the online version of the article.

We apologize for this error.

